# Evaluation of pericardium patch graft thickness in patients with Ahmed glaucoma valve implantation: an anterior segment OCT study

**DOI:** 10.1007/s10384-024-01051-9

**Published:** 2024-03-30

**Authors:** Yusuf Berk Akbas, Nese Alagoz, Cem Sari, Cigdem Altan, Tekin Yasar

**Affiliations:** 1 Department of Ophthalmology, University of Health Sciences, Basaksehir Cam and Sakura City Hospital, Istanbul, Turkey; 2 Department of Ophthalmology, University of Health Sciences, Beyoglu Eye Training and Research Hospital, Istanbul, Turkey

**Keywords:** Ahmed Glaucoma Valve, Anterior segment optical coherence tomography, Exposure, Pericardium patch graft, Glaucoma drainage devices

## Abstract

**Purpose:**

To evaluate the changes in thickness of tissues, specifically the pericardium patch graft (PPG) covering the silicone tube in Ahmed Glaucoma Valve (AGV) surgery.

**Study design:**

Prospective observational study.

**Methods:**

This study included cases with refractory glaucoma that underwent AGV implantation with PPG coverage. Conjunctival epithelium, stroma and PPG thickness covering the tube were measured using anterior segment optical coherence tomography (AS-OCT) at 1, 6 and 12 months. Additionally, the same measurements were taken 1500 µm away from the tube as a control for the central measurements.

**Results:**

Twenty-seven eyes of 27 patients were evaluated in the study. Although PPG thickness decreased significantly in both regions, the amount of reduction was more pronounced centrally. Centrally, the reduction rate was 21.2% and 34.8% during the 1-6 months period and 6-12 months period, while peripherally it was 3.5% and 5.1%, respectively. No change was observed in the thickness of the epithelium during the follow-up period. There was a significant thinning of the stroma in the central and peripheral regions during the 1-6 months period (30.5% and 17%, respectively). No cases of exposure were observed during the follow-up period.

**Conclusion:**

Although the most evident thinning of the layers covering the tube was observed in the early postoperative period, PPG showed a stable decrease even in the late period. The progressive reduction in the PPG thickness observed also in the peripheral region indicates that factors beyond mechanical forces contribute to this degenerative process. AS-OCT could be a valuable non-invasive tool in clarifying this process.

## Introduction

Glaucoma drainage device implantation is a choice in refractory glaucoma [1–3]. The devices function by creating an aqueous shunt between the anterior or posterior chamber and subconjunctival space. The Ahmed Glaucoma Valve® (AGV) (New World Medical) consists of a reservoir plate attached to a tube with a flow restrictor system to avoid postoperative hypotony [4]. In the proximal part of the reservoir, there is a valve system which opens when intraocular pressure exceeds 12 mmHg and closes when it drops below 7 mmHg [5, 6].

Exposure of the tube is a serious postoperative complication of AGV implantation that may result in sight-threatening infections [7–9]. To minimize the risk of tube exposure, several surgical techniques have been described, including various patch graft materials, Tenon’s duplication and long scleral tunneling [10–13]. The commonly used graft materials include dura mater, cornea, sclera, fascia lata, amniotic membrane and pericardium.

Although these precautions reduce the risk of tube exposure, conjunctival erosions can occur in patients with glaucoma drainage implants [9, 14, 15]. This prospective study aimed to quantitatively evaluate the changes in thickness of the layers covering the tube, including the pericardium patch grafting (PPG), Tenon's layer, and conjunctiva, using anterior segment optical coherence tomography (AS-OCT) in patients who underwent AGV implantation combined with PPG.

## Materials and methods

This prospective study was conducted in the glaucoma clinic of a tertiary eye hospital following approval granted by the institutional review board and the local ethics committee (University of Health Sciences Ethics Committee, reference number: E-46418926-050.01.04—11132). Written informed consent was provided by all subjects in accordance with the principles of the Helsinki Declaration. The study included consecutive patients diagnosed with refractory glaucoma who underwent AGV implantation with PPG coverage (Tutoplast^®^, IOP) between January 2020 and January 2021. Inclusion criteria were patients over 18 years of age who had completed 12 months of follow-up. Patients who underwent glaucoma drainage device implantation other than AGV, patients with AGV implantation through scleral tunnel or who received AGV implantation in a quadrant other than the superotemporal quadrant, were not included in the study. Patients with insufficient quality of AS-OCT images restricting the visualization of all 3 layers and patients who did not comply with the follow-up schedule were excluded.

The surgical technique for AGV implantation consisted of a superotemporal fornix-based conjunctival incision, blunt dissection of Tenon’s capsule, implantation of the body to 10 mm posterior to the limbus with a Dacron suture, trimming the end of the tube to an approximately 30 degree bevel-up shape, anterior chamber (AC) paracentesis and viscoelastic injection, entry into the AC 2 to 3 mm posterior to the limbus with a 23 G needle, insertion of the tube into the AC, patching the tube with PPG fixed to the sclera at four corners with 10-0 nylon sutures, and finally closure of the conjunctiva and Tenon’s layer with 10-0 nylon sutures. In eyes with a previous trabeculectomy surgery the AGV implant site was different from the previous trabeculectomy region. All eyes received topical moxifloxacin five times daily for one week, topical prednisolone acetate six times daily for one week and tapered in four weeks. In addition, topical antiglaucoma medications or mydriatics were added as needed during follow-up.

At each visit, all patients underwent a detailed ophthalmic examination including measurement of corrected distance visual acuity (CDVA) with Snellen charts, biomicroscopic examination of the anterior segment, measurement of intraocular pressure (IOP) with Goldmann tonometry and a dilated posterior segment examination. Demographic data and topical medications were also recorded. All patients were examined postoperatively at 1 day, 1 week, 1, 3, 6 and 12 months. AS-OCT images were obtained at 1, 6 and 12 months to evaluate the thickness of the layers covering the tube. Images obtained earlier than 1 month did not allow for proper identification of the layer boundaries covering the tube. Therefore, the measurement at 1 month was considered as the reference thickness to compare with the measurements at 6 and 12 months in the current study.

High-resolution AS-OCT images of the distal tube portion of the AGV implant were acquired with the anterior segment module of the Spectralis OCT imaging device (Heidelberg Engineering) which is a 40-kHz spectral-domain OCT with a center wavelength of 870 nm. An adaptive add-on lens was used. All patients were asked to look inferomedially to a standard fixation point, and the upper eyelid was gently retracted to view the area with PPG while the measurements were taken. At least 3 measurements were obtained in horizontal sections using the sclera module of the device.

**Fig. 1:**
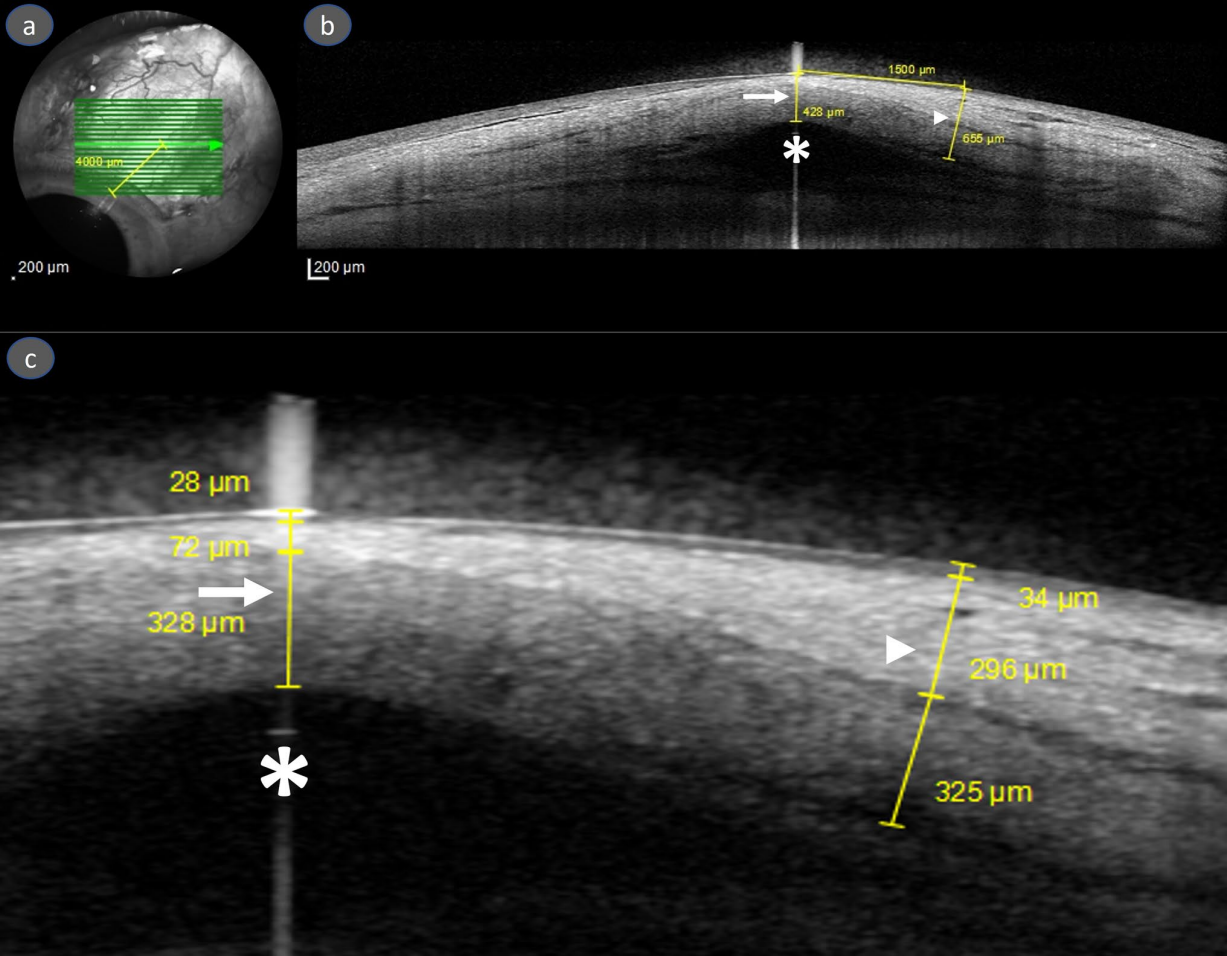
Measurements performed in central and peripheral regions. For all measurements the image 4000 μm (4 mm) from the limbus was selected (**A**). The central measurement (arrowhead) of total thickness was performed to the layers covering the top of the silicone tube (asterisk shows the center of the tube) and the peripheral measurement (arrow) of total thickness was performed to the same layers 1500 μm temporal to the center of the tube (**B**). Measurements of each layer in central and peripheral region (from inner to outer: pericardial patch graft, stroma and bulbar conjunctival epithelium) (**C**)

The evaluation of the OCT images was performed by two independent blind examiners (YBA and CS) and the mean of the two measurements was recorded. The measurement of the layers was provided for 2 different locations: the central and peripheral which corresponded respectively to the region covering the tube itself and the region that is not related to and is therefore away from the tube. The peripheral measurement was used as a control. All measurements were performed on the same OCT image. For the standardization and accuracy of the measurements the image 4000 μm (4 mm) from the limbus (Fig. 1A) was selected for all examinations, including the baseline and follow-ups. The central measurement was performed to the layers covering the top of the silicone tube and the peripheral measurement to the same layers 1500 μm temporal to the center of the tube (Fig. 1B). It was noted that the border between the conjunctival epithelium and conjunctival stroma and the border between PPG and the Tenon’s layer were clearly delineated, but the border between the conjunctival stroma and the Tenon’s layer was not. Therefore, the conjunctival stroma and Tenon’s layer were evaluated as a whole and together referred to as stroma in the present study, resulting in 3 different thickness measurements: Bulbar conjunctival epithelium, the stroma and PPG (Fig. 1C).

Statistical analysis was performed using SPSS for Windows (version 22.0, SPSS, Inc.). Visual acuity measurements were converted to LogMAR units for statistical purposes. Continuous variables were expressed as the mean and standard deviation. The Shapiro–Wilk test was used to assess the normality of the data; Friedman and Wilcoxon tests were used to compare postoperative measurements. P values of <0.05 were considered statistically significant. Pairwise comparisons were made at 1, 6 and 12 months using the Bonferroni correction method and p values of <0.016 were considered statistically significant.

## Results

### Demographics and clinical characteristics

A total of 33 eyes of 33 patients that received AGV implantation with PPG were recruited into the study. Due to the poor image quality in four eyes and two patients who were lost to follow-up, 27 eyes of 27 patients were included in the analysis. Table 1 summarizes the demographic and clinical characteristics of the patients. The mean age of the patients was 39.8±12.9 years and most of the patients (77.7%) were diagnosed with secondary glaucoma. AGV implantation was the initial glaucoma surgery in 6 of the eyes, while 21 eyes had undergone a previous glaucoma surgery (Table 1).

**Table 1:** Demographics and clinical characteristics of the patients

Patient data	Total (*n*=27)
Demographics	
Age (min-max), years	39.84 ± 12.9 (23–75)
Sex ratio, M/F	19/8
Caucasian	27
Lens Status	
Phakic	10
Pseudophakic	16
Aphakic	1
Preoperative clinical characteristics	
Preoperative visual acuity, LogMAR	1.52 ± 0.88
The number of preoperative topical anti-glaucomatous drugs	3.38 ± 0.64
Preoperative IOP, mmHg Diabetes	34.7 ± 9.8 6 (22.2%)
Glaucoma classification	
Primary open angle	4 (14.8%)
Congenital	2 (7.4%)
Traumatic	5 (18.5%)
Uveitic	4 (14.8%)
Silicone oil induced	4 (14.8%)
Neovascular glaucoma	4 (14.8%)
Post-penetrating keratoplasty	2 (7.4%)
İridocorneal endothelial syndrome	1 (3.7%)
Axenfeld-Rieger anomaly	1 (3.7%)
Previous glaucoma surgery	
None	6 (22.2%)
Trabeculectomy	9 (33.3%)
Cyclophotocoagulation	14 (51.9%)
Postoperative anti-glaucomatous drugs	
Brimonidine Dorzolamide+Timolol Latanoprost	10 (37.0%) 20 (74.1%) 5 (18.5%)

### AS-OCT measurements

Table 2 shows the mean conjunctival epithelium, stroma and PPG thickness measurements in the central and peripheral regions using AS-OCT at 1, 6 and 12 months postoperatively.

**Table 2:** Central and peripheral thickness measurements on AS-OCT

	Month 1	Month 6	Month 12	P^1^	P^2^	
Central region						
Epithelium (μm)	45.9±20.9	37.9±15.9	36.9±12.2	0.145	Months 1–6	0.057
					Months 6–12	0.669
					Months 1–12	0.097
Stroma (μm)	151.4±74.5	105.2±61.4	106.1±55.1	0.001	Months 1–6	0.015
					Months 6–12	0.334
					Months 1–12	0.001
PPG (μm)	225.0±61.6	177.3±74.5	115.6±47.2	<0.001	Months 1–6	0.001
					Months 6–12	0.001
					Months 1–12	<0.001
Total thickness (μm)	422.3±111.2	320.4±110.7	258.6±89.4	<0.001	Months 1–6	0.001
					Months 6–12	0.002
					Months 1–12	<0.001
Peripheral region						
Epithelium (μm)	62.6 ± 30.8	66.3 ± 26.3	58.4 ± 18.4	0.247	Months 1–6	0.744
					Months 6–12	0.460
					Months 1–12	0.193
Stroma (μm)	278.8 ± 100.7	231.3 ± 97.3	235.4 ± 98.3	0.047	Months 1–6	0.043
					Months 6–12	0.256
					Months 1–12	0.018
PPG (μm)	349.5 ± 60.3	337.2 ± 40.3	319.9 ± 55.8	<0.001	Months 1–6	0.001
					Months 6–12	0.023
					Months 1–12	0.003
Total thickness (μm)	690.9 ± 132.7	634.8 ± 123.1	613.7 ±115.4	<0.001	Months 1–6	0.006
					Months 6–12	0.020
					Months 1–12	0.001

*AS-OCT *Anterior segment optic coherence tomography, *PPG* pericardial patch graft

P^1^ values were obtained using Friedman test

P^2^ values were obtained using Wilcoxon signed-rank test

In both central and peripheral regions, no significant change in mean conjunctival epithelium thickness was observed during the follow-up period. However, a significant decrease in mean stroma and PPG thickness was observed. During the 1-6 month period the stroma thickness was found to decrease significantly in the central region (p=0.015) and non-significantly in the peripheral region (p=0.043), and remained stable thereafter (Table 2). Central stromal thickness change was 30.5% during the 1-6 month and 0.9% during the 6-12 month period. The peripheral stromal thickness change was 17% during the 1-6 month and 1.79% during the 6-12 month period (Table 3). On the other hand, the thickness of PPG progressively decreased throughout the entire study period in both regions (Table 2). The percentage of reduction was 21.2% and 34.8% centrally and 3.5% and 5.1% peripherally in the 1-6 month and 6-12 month periods, respectively. The reduction in PPG thickness was more prominent in the central region (Table 3).

**Table 3:** Thickness loss in Stroma, PPG and total thickness observed in different periods

	1-6 months period	6-12 months period	1-12 months period
Central region			
Stroma [µm (%)]	46.2 ± 17.4 (30.5%)	− 0.9 ± 1.7 (0.9%)	45.3 ± 11.3 (29.9%)
PPG [µm (%)]	47.7 ± 8.0 (21.2%)	61.7 ± 5.7 (34.8%)	109.4 ± 91.7 (48.6%)
Total Thickness [µm (%)]	101.9 ± 98.5 (24.1%)	61.8 ± 51.8 (19.3%)	163.7 ± 107.9 (38.7%)
Peripheral region			
Stroma [µm (%)]	47.5 ± 21.3 (17%)	− 4.1 ± 3.2 (1.7%)	43.4 ± 36.6 (15.6%)
PPG [µm (%)]	12.3 ± 34.1 (3.5%)	17.3 ± 25.9 (5.1%)	29.6 ± 31.2 (8.4%)
Total Thickness [µm (%)]	56.1 ± 61.9 (8.1%)	21.1 ± 23.9 (3.3%)	77.2 ± 70.3 (11.1%)

*PPG* pericardial patch graft

**Fig. 2:**
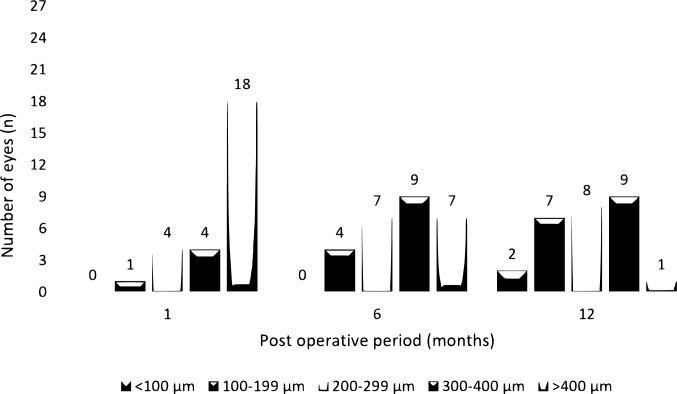
Distribution of the total thickness in central region throughout the study period.

The total thickness of the layers decreased centrally from 422.3±111.2 μm at 1 month to 258.6±89.4 μm at 12 months, resulting in a 163.7 μm reduction and 38.7% change. Peripherally, the thickness decreased from 690.9±132.7 μm at 1 month to 613.7±115.4 μm at 12 months, indicating a 77.2 μm reduction and 11.1% change (Tables 2 and 3). Tube exposure was not observed in any of the patients during follow-up, but two (7.4%) of the eyes were measured to have a total thickness of less than 100 μm (79 μm and 86 μm) in the central region at 12 months. Figure 2 shows the central thickness distribution over the follow-up visits.

In the subgroup analysis of cases with previous trabeculectomy surgery, cyclophotocoagulation, and naïve eyes, there was no statistically significant difference when comparing the changes in the PPG and total thickness in both the central and peripheral regions. Furthermore, the evaluation of the factors contributing to postoperative conjunctival thinning revealed a moderate correlation between the total number of anti-glaucoma eye drops and reduction in the total peripheral thickness (r=0.446, p=0.02). No association was found between the presence of diabetes mellitus, hypertension, type of antiglaucoma medication, previous surgery, or glaucoma etiology and conjunctival thinning.

### Other clinical findings

Table 4 shows the change in the mean IOP, number of glaucoma medications and CDVA.

**Table 4:** Change in mean IOP, number of glaucoma medication and BCVA

	Preoperative	Month 1	Month 6	Month 12	P
IOP (mmHg)	34.69 ± 9.82	17.12 ± 8.04	13.85 ± 5.70	13.27 ± 4.55	<0.001
The number of glaucoma medication (*n*)	3.38 ± 0.64	1.35 ± 1.26	2.12 ± 1.28	2.31 ± 1.26	<0.001
BCVA (LogMAR)	1.52 ± 0.88	1.43 ± 0.92	1.41 ± 0.97	1.44 ± 0.97	0.089

*IOP* Intraocular pressure, *BCVA* Best corrected visual acuity

*P* values performed using Friedman test, *P* < 0.05 was accepted as statistically significant.

## Discussion

Glaucoma drainage device implantation is a surgical option for patients with refractory glaucoma. Although various graft materials are used, the rate of tube exposure after seton implantation ranges from 2 to 7% [16–18]. Tube exposure is a significant risk factor for endophthalmitis which currently occurs at rates ranging from 0.00197% to 6.3% [19]. There are various theories for the development of tube exposure; the tissues covering the tube may be affected by inflammatory, ischemic, and mechanical factors. Mechanical factors such as friction due to upper eyelid movements during blinking and direct pressure from the tube play a significant role [20], while conjunctiva and Tenon’s layer compression might lead to ischemia in these tissues [21]. In addition, anti-glaucoma eye drops could potentially cause thinning of the conjunctiva [22, 23].

Several techniques have been developed to prevent tube exposure. Materials such as cornea, sclera, amniotic membrane, fascia lata and pericardium may be utilized to cover and strengthen the overlay. However, in certain cases, these materials prove insufficient to prevent exposure [7, 20]. Previous studies identify risk factors for tube exposure including female sex, older age, Hispanic ethnicity, inferior placement of the device, neovascular glaucoma, and previous intraocular surgery [24–27].

In the current study, we aimed to assess the rate of thickness reduction in the tissues covering the tube. For this purpose, we prospectively followed-up the thickness changes in the layers covering the tube in eyes that had undergone AGV implantation with PPG coverage. AS-OCT was used to measure the thickness of conjunctival epithelium, stroma and PPG separately. To the best of our knowledge, this is the first study that measured the thickness of different tissues overlying the tube quantitatively using an AS-OCT.

In the present study, the thickness of the epithelial layer showed a non-significant decrease in the central region and a more stable trend in the peripheral region. Central thinning may have been caused by mechanical pressure of the tube or epithelial remodeling that compensated for the elevation of the layers over the tube. The thinning of the corneal epithelial layer is a well-documented phenomenon in regions where the cornea is elevated, such as in keratoconus [28]. However, there is no report on the behavior of the conjunctival epithelium. Furthermore, the stromal layer’s thickness decreased in the early postoperative period and remained unchanged. Centrally, the rate of reduction in the stromal thickness was 30.5% and 0.9%, peripherally it was 17% and 1.7% in the 1-6 months and 6-12 months period, respectively. The reduction in stromal thickness, which only occurs in the early postoperative period, is thought to be caused by the resolution of postoperative inflammation and edema. The greater decrease in thickness in the center could potentially be due to the mechanical pressure of the tube.

PPG thickness, on the other hand, demonstrated a continuous thinning throughout the study period. Centrally, the mean PPG thickness decreased from 225 μm at 1 month to 177 μm at 6 months and to 115 μm at 12 months. Peripherally PPG decreased from 349 μm at 1 month to 337 μm at 6 months and to 319 μm at 12 months. Like the stromal layer, the PPG layer was thinner in the center at all examinations, reflecting the effect of compression of the tissue by the tube itself. There was a significant difference also in the rate of reduction between the two regions. The reduction in PPG thickness in the central and peripheral regions was 21.2% vs. 3.5% in the 1-6 months period and 34.8% vs. 5.1% at 6-12 months period. The fact that the thinning rate in the central region was higher was consistent with the mechanical factor theory of exposure. Ainsworth et al. also postulate that increased tension on the conjunctiva overlying the tube leads to mechanical breakdown of the tissues [20]. Additionally, the eyelid margin constantly rubbing against the tissue patch graft and conjunctival desiccation contribute to the mechanical factor theory [7, 29].

Gradual thinning of the graft over time could be related to a low-grade immune-mediated response [15]. However, the Tutoplast^®^ PPG is processed through gamma irradiation and chemical dehydration, hence it is expected to be cell-free and without antigenic stimuli [7]. Thus, the thinning of PPG could be due to non-cell mediated immune responses. In a previous study, it was demonstrated that dura and fascia lata xenografts were enzymatically broken down by the surrounding granulation tissue in albino rats [30]. The histologic sections of the grafts displayed collagen breakdown, fibroblast proliferation and collagen fibril replacement which occurred without any sign of acute inflammation. In our study, even though the central thinning of the layers was prominent, the peripheral thickness of PPG also reduced in time. This proves that factors beyond mechanics contribute to the thinning of PPG in the periphery even where there is no pressure or compression on the layers. However, future studies are needed to clarify the exact mechanisms of the progressive PPG thinning in the human eye.

Studies lack a definitive consensus on the most exposure-resistant material among the various patch materials. Smith et al. compared dura mater, sclera and pericardium for graft thinning, evaluated by inspection [15]. They report thinning in 26% of the grafts over two years without any significant difference between the groups [15]. Similarly, exposure rates were not found to be different between donor dura and sclera allografts in one of the largest series published in the literature involving 1816 procedures [31]. Moreover, another study comparing Tutoplast^®^ pericardium, Tutoplast^®^ sclera and eye bank sclera found no association between the likelihood of exposure and the type of patch graft [32]. Yet some studies report inferior results with PPG when compared to other materials. Wigton et al. found that glycerol preserved corneal tissue significantly reduces the risk of exposure and prolongs the initial exposure time compared to PPG [33]. Sheeha et al. observed a higher incidence of early graft thinning (42.5%) and late progressive thinning (27.5%) in the pericardium group compared to the amniotic membrane-umbilical cord group [27].

Limitations of the current study include relatively low number of patients and heterogeneity of glaucoma classification. Although exposure was not observed in any patient throughout the study period, exposure rate is unknown beyond 12 months. Nevertheless, the study revealed important findings useful to clarify the course of tube exposure after pericardial patch grafting.

In conclusion, after the implantation of AGV with PPG, the mean thickness of PPG decreased throughout the 1-year follow-up both in central and peripheral regions. The thinning process was more pronounced centrally. The progressive reduction of the PPG thickness observed also in the peripheral region proved to us that factors besides the mechanical factors are involved in this degenerative process.

## Data Availability

The data that support the findings of this study are available from the corresponding author upon reasonable request.
